# Gambling disorder and suicidal behavior : A case report :

**DOI:** 10.1192/j.eurpsy.2022.2149

**Published:** 2022-09-01

**Authors:** B. Abassi, E. Khelifa, O. Maatouk, S. Ben Aissa, I. Bouguerraa, L. Mnif

**Affiliations:** 1 Razi, Skolly, Manouba, Tunisia; 2 Razi Hospital, F Adult Psychiatry Department, Manouba, Tunisia

**Keywords:** gambling, Addiction, behaviour, Suicide

## Abstract

**Introduction:**

Gambling disorder involves repeated problematic gambling behavior that causes significant problems or distress. It is also called gambling addiction or compulsive gambling.

In Tunisia, a muslum country, gambling is prohibited and casinos are non-existent or only for tourists with foreign currency. Lately, with the spread of casinos online and sites of sports betting, gambling’s become problematic in Tunisia. People accumulated huge debts with feelings of shame and guilt leading to suicidal attempts.

**Objectives:**

Studying the link between gambling disorder and suicidal attempts and comparing the different preventive measures proposed for online gambling.

**Methods:**

a case of a patient with gambling disorder that was hospitalized in a psychiatric hospital for a suicidal attempt by stabbing himself and a review of a literature.

**Results:**

Mr R.A was a 42-year-old man with no family nor personal psychiatric history. He has no history of a particular substance use disorder. He was married and a father of two children and has a regular job.

A year ago, he stated gambling on internet sites using his phone cell and, in several months, he lost a lot of money and accumulated debts.

Lately he committed two attempts of suicide. The first one was by swallowing rat poison. The second one was a month later, by stabbing himself with a knife that caused evisceration and required surgery then an hospitalization in a psychiatric unit.

**Conclusions:**

There’s evidence that GD and SB are associated, although there’s disagreement about the nature of this association. Adequate preventive measures should be considered by governments

**Disclosure:**

No significant relationships.

